# Threshold estimation based on local minima for nucleus and cytoplasm segmentation

**DOI:** 10.1186/s12880-022-00801-w

**Published:** 2022-04-26

**Authors:** Simeon Mayala, Jonas Bull Haugsøen

**Affiliations:** 1 Department of Mathematics, University of Bergen, Allégaten 41, 5007 Bergen, Norway; 2 Department of Clinical Medicine, Neuro-SysMed, University of Bergen, PO box 7804, 5020 Bergen, Norway; 3 Department of Neurology, Neuro-SysMed, Haukeland University Hospital, Jonas Lies vei 71, 5053 Bergen, Norway

**Keywords:** Segmentation, Local minima, Nucleus and cytoplasm

## Abstract

**Background:**

Image segmentation is the process of partitioning an image into separate objects or regions. It is an essential step in image processing to segment the regions of interest for further processing. We propose a method for segmenting the nuclei and cytoplasms from white blood cells (WBCs).

**Methods:**

Initially, the method computes an initial value based on the minimum and maximum values of the input image. Then, a histogram of the input image is computed and approximated to obtain function values. The method searches for the first local maximum and local minimum from the approximated function values in the order of increasing of knots sequence. We approximate the required threshold from the first local minimum and the computed initial value based on defined conditions. The threshold is applied to the input image to binarize it, and then post-processing is performed to obtain the final segmented nucleus. We segment the whole WBC before segmenting the cytoplasm depending on the complexity of the objects in the image. For WBCs that are well separated from red blood cells (RBCs), *n* thresholds are generated and then produce *n* thresholded images. Then, a standard Otsu method is used to binarize the average of the produced images. Morphological operations are applied on the binarized image, and then a single-pixel point from the segmented nucleus is used to segment the WBC. For images in which RBCs touch the WBCs, we segment the whole WBC using SLIC and watershed methods. The cytoplasm is obtained by subtracting the segmented nucleus from the segmented WBC.

**Results:**

The method is tested on two different public data sets and the results are compared to the state of art methods. The performance analysis shows that the proposed method segments the nucleus and cytoplasm well.

**Conclusion:**

We propose a method for nucleus and cytoplasm segmentation based on the local minima of the approximated function values from the image’s histogram. The method has demonstrated its utility in segmenting nuclei, WBCs, and cytoplasm, and the results are satisfactory.

**Supplementary Information:**

The online version contains supplementary material available at 10.1186/s12880-022-00801-w.

## Background

White blood cells (WBCs) are cells of the immune system that take part in the body’s defense against infectious disease and foreign material [1, 2]. WBCs can be categorized based on structure (granulocytes or agranulocytes) and cell lineage (myeloid or lymphoid cells). Broadly, there are five types of WBCs; three types of granulocytes—neutrophils, eosinophils, and basophils, and two types of agranulocytes—lymphocytes and monocytes. Granulocytes and monocytes are of myeloid lineage, whereas lymphocytes are, as the name implies, of lymphoid lineage.

In microscopic images of stained blood smears, WBCs can be differentiated from red blood cells and platelets by having nuclei and their (in most cases) larger size. They also stain darker using common dyes such as hematoxylin and eosin (H&E). Three main characteristics are used to identify the different types of WBCs - the shape of their nuclei, their granularity, and their staining.

**Fig. 1:**
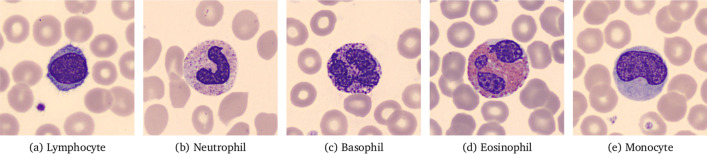
Normal white blood cells. The cells seen in the background are red blood cells identified by their red color, thin (often almost transparent) center, and lack of nuclei

All the granulocytes are large, granular cells with lobulated nuclei [2]. Neutrophils stain neutrally and have nuclei with multiple (2–5) lobes. Eosinophils stain red and have nuclei with 2–4 lobes. Basophils stain blue and have nuclei with 2–3 lobes. The shapes of these lobes are characteristic for each cell type, as seen in Fig. 1. Monocytes are large agranular cells with kidney-shaped nuclei. Lymphocytes are also agranular and are smaller than the other WBCs. Their nuclei are round, often eccentric, and stain dark blue. Examples of the different types of WBCs can be seen in Fig. 1.

Image segmentation is a process that separates a region of interest (ROI) from the background to simplify further analysis [3, 4]. Different methods for segmenting images have been developed. They are classified differently based on how they perform the segmentation. Example of the popular methods include thresholding [5–9], edge detection [10], morphologically based [11], graph based [12–14], clustering based [15], watershed [16], level-set based [17] and a combination of different methods [2, 18–20]. Convolutional neural networks (CNN) are deep learning techniques and they have been widely used for WBCs segmentation [21–23]. We review related literature concerning WBC and nucleus segmentation.

Mittal et al. [24] presents a comprehensive review of different computer-aided methods for analyzing blood smear images and leukemia detection. The paper reviews 149 papers by presenting different techniques used for preprocessing images and WBC segmentation methods. It provides different workflow pipelines for segmenting WBC based on knowledge, deformable models, and machine learning. Also, the review gives the merits and demerits of each method.

Li et al. [9] proposed a dual-threshold method for segmenting WBC based on a strategic combination of the RGB and HSV colour spaces by searching for an optimal threshold using a golden section search method. Ghane et al. [18], proposed a method for segmenting WBC based on a combination of thresholding, K-means clustering and a modified watershed algorithm. Also, Kuse et al. [19] proposed a method that segments cells using mean shift-based clustering for color approximation and then thresholding. Features are extracted and then used to train a support vector machine (SVM) classifier for classifying lymphocytes and non-lymphocytes. Prinyakupt et al. [2] proposed a system that pre-processes the images by locating the WBCs and then segmenting them into nucleus and cytoplasm.

Theera-Umpon [11] proposed a method that uses a fuzzy C-means (FCM) algorithm and mathematical morphological operations to segment WBCs. Miao and Xiao [16] proposed a marker-controlled watershed algorithm for segmenting WBCs and RBCs. Yung-Kuan et al. [10] propose a method for WBC nucleus segmentation and counting the lobes in a nucleus that works by object contour detection. Furthermore, Salem [15] proposed a WBC segmentation method based on the K-means clustering technique. The method converts RGB to the $$L*a*b$$ color space and then the *a* and *b* components are used as features in the clustering algorithm. Sadeghian et al. [20] propose a framework that integrates several digital image processing algorithms for segmenting nucleus and cytoplasm. Khamael et al. [17] propose a method for the segmenting nuclei of WBC from the cytoplasm and the cell wall. The method performs segmentation based on level set methods and geometric active contours.

Banik et al. [21] proposed a method that segments WBC nuclei based on a $$L*a*b$$ color space conversion and K-means algorithm. Then, WBCs are located using the segmented nucleus. A convolutional neural network (CNN) is used to classify the localized WBC image. Fan et al. [22] proposed a method for localization and segmentation of WBCs. The method uses pixel-level information for training a deep convolutional neural network to locate and segment the region of interest. Lu et al. [23] proposed a WBC-NET based on the UNet++ and ResNet to improve the accuracy of the WBCs segmentation. Furthermore, Long [25] proposed an enhanced, light-weighted U-Net with a modified encoded branch. The method explores the possibility of performance improvement of cell nucleus segmentation algorithms through deep learning, requiring less pre-and post-processing of images.

Our contribution in this work concerns the way the method estimates the threshold. A histogram of the input image is computed, and the function values are approximated. The threshold is estimated based on the local minima of the approximated function values. The estimated threshold is applied to the input image to segment the nucleus. Also, we develop a simple strategy for segmenting the WBC whenever is well separated from surrounding red blood cells. We generated *n* different thresholds and each threshold is applied to the input image to produce *n* thresholded images. The produced images are combined by taking their average and then a standard Otsu method is used to binarize it. We perform post-processing and use a single-pixel point from the nucleus to extract the WBC. The cytoplasm is obtained by subtracting the segmented nucleus from the segmented WBCs.

For images whose WBCs touch RBCs, we opt for classical techniques to separate the touching objects. We use the Simple Linear Iterative Clustering (SLIC) approach based on superpixels [26]. Since the focus is on the WBCs, we utilize the superpixel’s strength of boundary adherence to segment the WBCs. We also apply a watershed transformation to segment the WBC [27]. The number of local maxima is chosen automatically. When the WBC is detached from the uninteresting objects, we perform post-processing so that only the WBC remains.

## Methods

**Fig. 2:**
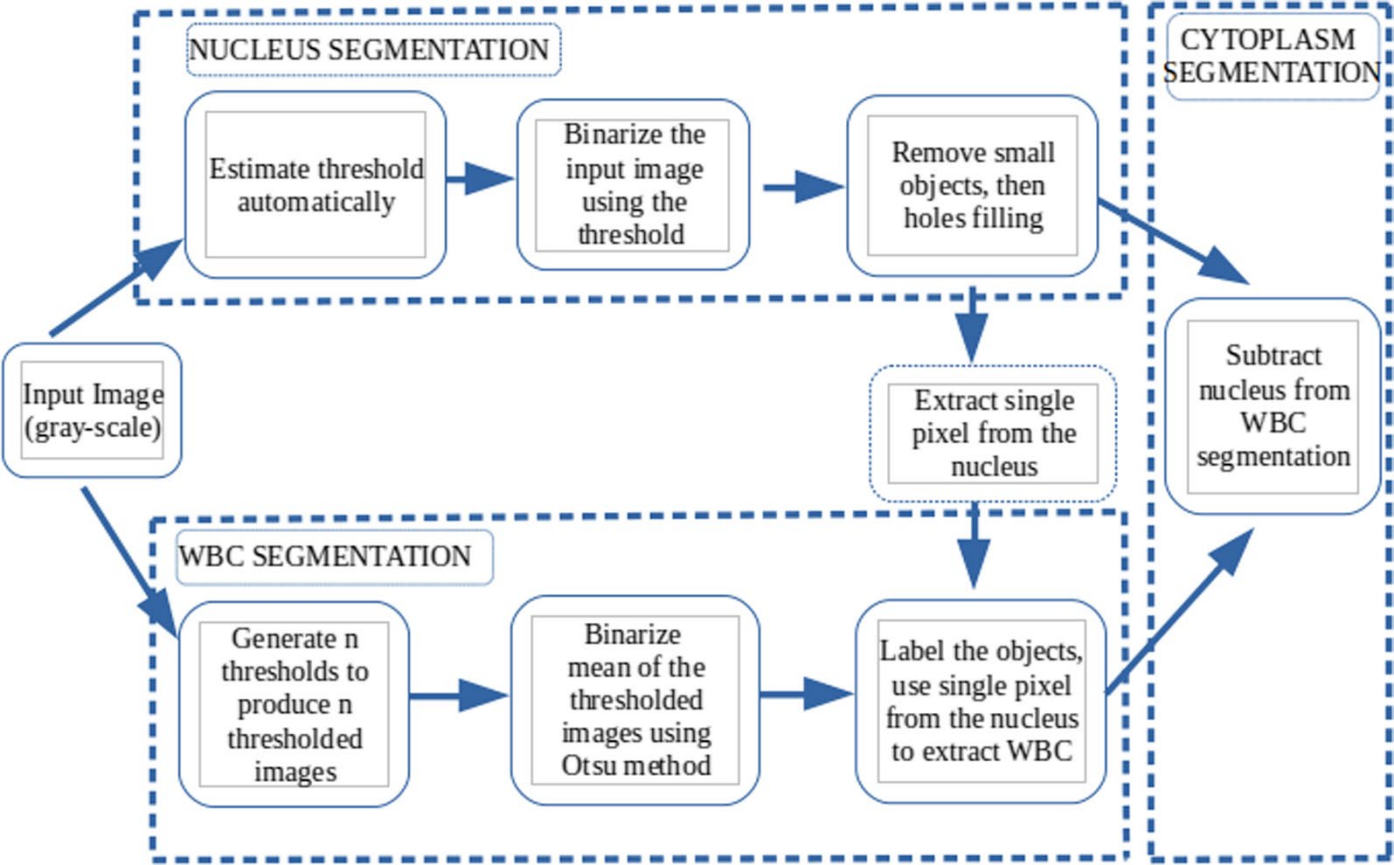
Schematic diagram. A block diagram summarizing the steps for nucleus, White blood cells, and cytoplasm segmentation

In this section, we establish a method for segmenting the nucleus and cytoplasm based on the distribution of the intensity values of the input image. The technique estimates the threshold automatically based on the local minima of the estimated function values. The summarized steps for nucleus, WBC, and cytoplasm segmentation are visualized in Fig. 2.

### Threshold estimation for nucleus segmentation

Assuming that a gray-scale image has intensity values that   can be classified into several gray levels. Let a gray-scale image *A* be a   matrix   of $M \times N$ dimension such that *f*(*x*, *y*) gives an intensity value at position (*x*, *y*). We define,

1 $$\begin{aligned} \begin{aligned} A = \begin{bmatrix} f(x,y) \\ \end{bmatrix}_{M \times N} \text { for } x = 0,1,\dots N-1; y = 0, 1, \dots M-1 \end{aligned} \end{aligned}$$

Considering the input image *A* in Eq. 1, we compute a value $T_{nc0}$ that will be used to search for a threshold value for segmenting the nucleus. The $T_{nc0}$ value is computed from the input image by

2 $$\begin{aligned} T_{nc0}(n_{1}, n_{2}) = \frac{Max(A)}{n_{1}} +\frac{Min(A)}{n_{2}} \end{aligned}$$

Where $n_{1}$ and $n_{2}$ are integers greater than 0. A simple analysis on how to choose $n_{1}$ and $n_{2}$ is presented in Additional file 1. Assuming that $T_{nc0}$ is an intensity value on the histogram of the input image *A*. We want to estimate $T_{nc0}$ such that $Min(A)<T_{nc0}<Max(A)$. We represent the input image *A* in a form that increases monotonically to locate the value $T_{nc0}$. Let ${\tilde{A}}$ be a vectorized matrix of *A*, then the intensity values *f*(*x*, *y*) at each position (*x*, *y*) in the vectorized matrix are

3 $$\begin{aligned} \small {\tilde{A}} = \begin{bmatrix} f(x,y)_{0}, f(x,y)_{1},f(x,y)_{2},\dots , f(x,y)_{k-2}, f(x,y)_{k-1} \end{bmatrix}^{T} \end{aligned}$$

Where *k* is the total number of pixels in the image *A* and *T* is a transpose. We construct a non-decreasing sequence of values in ${\tilde{A}}$ by using the intensity values   *f*(*x*, *y*) at each position in ${\tilde{A}}$.  Then, the sequence will be

4 $$\begin{aligned} \small {\tilde{A}}_{seq} = \begin{bmatrix} f(x,y)_{i}\le f(x,y)_{i+1}\le \dots \le f(x,y)_{k-2} \le f(x,y)_{k-1} \end{bmatrix} \end{aligned}$$

The constructed sequence of intensity values in ${\tilde{A}}_{seq}$ are ordered sets and preserve a non-decreasing order, so it takes the form of a monotonically increasing function. For each $f(x,y) \in {\tilde{A}}_{seq}$ we can write $f(x,y)_{i}\rightarrow I_{i}$. The form has been changed from the function of two variables *f*(*x*, *y*) in image *A* into the function of one variable in ${\tilde{A}}_{seq}$

5 $$\begin{aligned} {\tilde{A}}_{seq} = \begin{bmatrix} I_{i}\le I_{i+1}\le \dots \le I_{k-2}\le I_{k-1} \end{bmatrix}= \begin{bmatrix} I_{i} \dots , T_{nc0}, \dots , I_{k-1} \end{bmatrix} \end{aligned}$$

$T_{nc0}$ is not necessarily one of the values in ${\tilde{A}}_{seq}$ but it is within the range of ${\tilde{A}}_{seq}$. A threshold ${\tilde{\varepsilon }}_{t}$ will be estimated after representing the intensity values of the input image in the form of a histogram. $T_{nc0}$ together with some conditions will be used to approximate the required threshold value.

### Approximating threshold ${\tilde{\varepsilon }}_{t}$

**Fig. 3:**
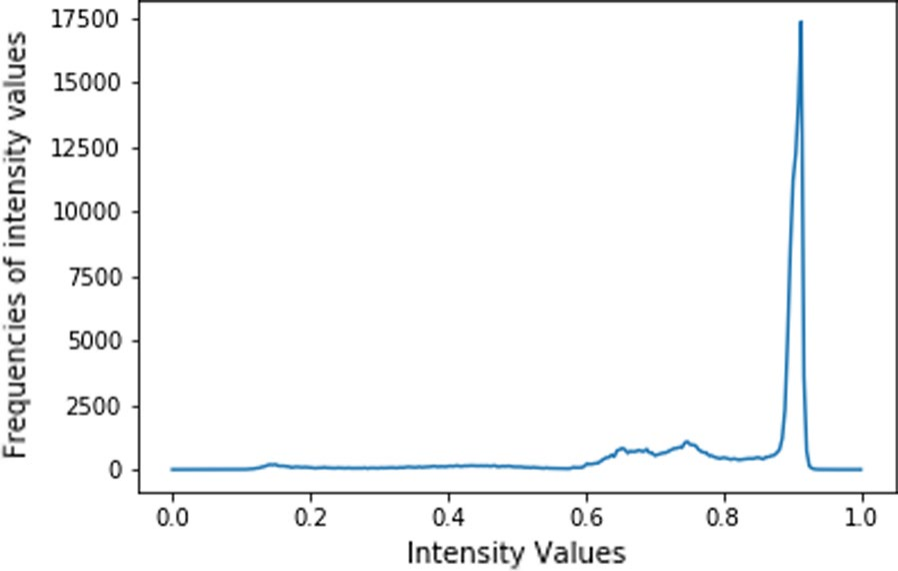
Histogram. Histogram of the input image represented by the function *h*(*x*)

**Fig. 4:**
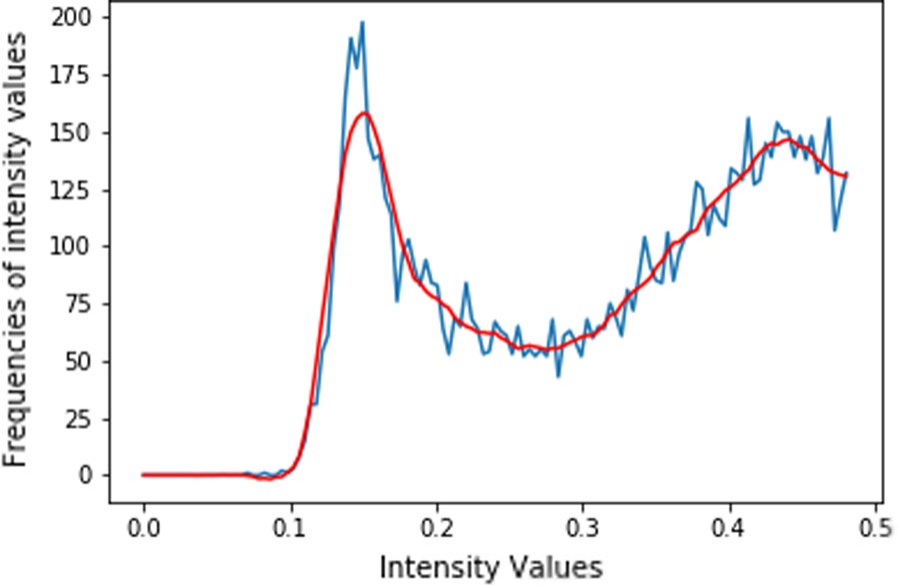
Estimating histogram. The smooth line represents $\tilde{h_{1}}(x)$, and the unsmooth line represents the original data points $h_{1}(x)$

Let *h*(*x*) be the function representing the histogram of an image *A*, where *x* is an intensity value *I*, and *h*(*x*) is the frequency of the intensity values (see Fig. 3). Let ${\tilde{h}}(x)$ be the approximation of *h*(*x*) (see Fig. 4). We approximate *h*(*x*) without distorting the general tendency of the histogram function. We can decompose ${\tilde{h}}(x)$ into

6 $$\begin{aligned} {\tilde{h}}(x) = \tilde{h_{1}}(x) + \tilde{h_{2}}(x) \end{aligned}$$

Let $\left( x_{i}, \tilde{h_{1}}(x_{i})\right) _{i=1}^{m}$ be the given points from the approximated histogram. We need to compute a spline *g* such that

7 $$\begin{aligned} g(x_{i}) = \tilde{h_{1}}(x_{i}), \text { for } i= 1,\dots , m \end{aligned}$$

The spline concept and basis spline (B-spline) is presented in Additional file 1 [28, 29]. Assuming that the spline is smooth and we are interested in the number of turning points produced from the spline *g*(*x*). We utilize the local propagation of B-Spline to choose any degree we want. So, a *g*(*x*) polynomial of *n* degrees gives $n-1$ turning points. Setting *d* be the degree of *g*(*x*), the turning points will be $\left( x_{1}, g(x_{1})\right)$, $\left( x_{2}, g(x_{2})\right)$, $\dots$, $\left( x_{d-1}, g(x_{d-1})\right)$.

**Fig. 5:**
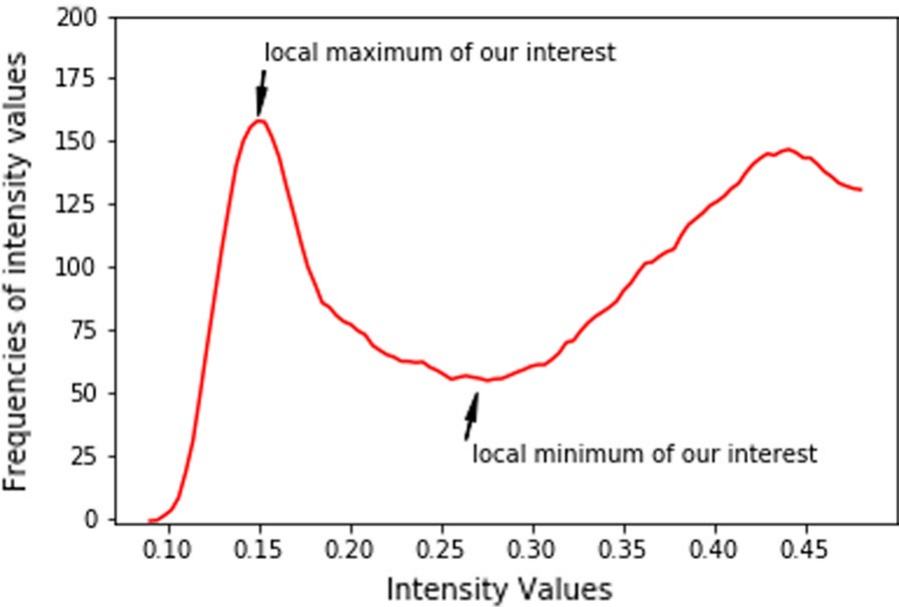
Estimated histogram. Figure showing the approximation $\tilde{h_{1}}(x)$ after removing the long flat tail. It shows the turning point (local minimum and maximum of interest)

Let $(x_{c}, g(x_{c}))$, $(x_{c+1}, g(x_{c+1}))$ be first local maximum and minimum of the function *g*(*x*) respectively. Based on the filtered data points of Fig. 5, the threshold ${\tilde{\varepsilon }}_{t}$ is expected to be closer to the first local minimum of the function *g*(*x*). Also, the threshold ${\tilde{\varepsilon }}_{t}$ must appear after the first local maximum of the function *g*(*x*) in the order of increase of the knots.

Also, from Eq. 2, the sensitivity analysis of $T_{nc0}$ in the Additional file 1 guarantees that the value of $T_{nc0}$ will not be very far from $\frac{1}{3}$. We introduce a parameter *Er* which is a small value chosen to control $T_{cn0}$ and the local minimum value during threshold estimation. Then, the threshold ${\tilde{\varepsilon }}_{t}$ is computed by checking the following conditions:

$$\begin{aligned} \text {(a) }&x_{c}< T_{nc0}.\\ \text {(b) }&\text {If }|T_{nc0}-x_{c+1}|<Er \text { then, }{\tilde{\varepsilon }}_{t} = \frac{T_{nc0} + x_{c+1}}{2}.\\ \text {(c) }&\text {If } |T_{nc0}-x_{c+1}|>Er \text { and } T_{nc0}>x_{c+1} \text { then, }{\tilde{\varepsilon }}_{t} = \frac{T_{nc0} + x_{c+1}+Er}{2}.\\ \text {(d) }&\text {If } |T_{nc0}-x_{c+1}|>Er \text { and } T_{nc0}<x_{c+1} \text { then, } {\tilde{\varepsilon }}_{t} = \frac{T_{nc0} + x_{c+1}-Er}{2}. \end{aligned}$$

The threshold ${\tilde{\varepsilon }}_{t}$ is applied on the input image *A* to produce image $A_{recons}$. We call it a reconstructed image, and it is mathematically obtained by

$$\begin{aligned}A_{recons} = {\left\{ \begin{array}{ll} Min(A) \text { if } f(x,y)\le {\tilde{\varepsilon }}_{t} \\ f(x,y) \text { elsewhere } \end{array}\right. }\end{aligned}$$

The image $A_{recons}$ is binarized to segment the nucleus of the WBCs. Then, post-processing operations are applied to the binarized image to obtain the final segmented nucleus.

### Threshold estimation for WBC segmentation

We develop a strategy for segmenting the WBC after preprocessing the input image. The input image is preprocessed by averaging *n* thresholded images of the input image to reduce the variability of intensity values in the WBC region. The *n* images are obtained using different thresholds produced in a defined range by using

8 $$\begin{aligned} T_{wbc}^{i}&= \frac{Max(A)+ T_{nc0}}{2} + er_{i} \end{aligned}$$

where $er_{i}$ is a step size between consecutive thresholds in a defined interval, $i = 0,1,2,\dots , n-1$.

Let $a_{l}$ and $a_{u}$ be lower and upper limit of the intensity values in the chosen interval respectively. We denote $er_{i}$ by $\bigtriangleup h$. Then, $\bigtriangleup h$ is a step size between two consecutive thresholds $T_{wbc}^{i}$ and $T_{wbc}^{i+1}$ in the interval between $a_{l}$ and $a_{u}$. To generate *n* equal sub-intervals in the defined interval we use

9 $$\begin{aligned} n = \frac{a_{u}- a_{l}}{\bigtriangleup h} \end{aligned}$$

as $\bigtriangleup h \rightarrow 0$, $n \rightarrow \infty$ and $(a_{u}- a_{l})$ is fixed. $T_{wbc}^{i}$ will be generated at the interval $\bigtriangleup h$. So, for the interval $a_{l}\le er_{i}\le a_{u}$ divided into $n-1$ equal subintervals is represented as

10 $$\begin{aligned} a_{l}= T_{wbc}^{0}< T_{wbc}^{1}<T_{wbc}^{2}<\dots <T_{wbc}^{n-1}=a_{u} \end{aligned}$$

where $T_{wbc}^{i}= a_{l}+i\bigtriangleup h$, for $i=0,1,2,\dots , n-1$ and $\bigtriangleup h = \frac{a_{u}-a_{l}}{n}$. Each $T_{wbc}^{i}$ is used to produce one thresholded image from the input image *A* by

11 $$\begin{aligned} A_{j} = {\left\{ \begin{array}{ll} T_{wbc}^{i} \text { if } f(x,y)\le T_{wbc}^{i} \\ f(x,y) \text { elsewhere } \end{array}\right. } \end{aligned}$$

Since *i* varies from 0 to $n-1$, then the reconstructed (preprocessed) image for WBC is produced by

12 $$\begin{aligned} A_{wbc} = \frac{1}{n} \sum _{j=0}^{n-1} A_{j} \end{aligned}$$

The image $A_{wbc}$ reduces the contrast in the WBCs region and the plasma membrane of the RBCs. The image $A_{wbc}$ is binarized to segment the WBCs from RBCs and other background. Note that the strategy works well for images whose WBCs are well separated from RBCs as well as the choice of *n*. For images whose WBCs touch the RBCs, we opt for classical techniques to segment the WBCs. The cytoplasm is obtained by subtracting the segmented nucleus from the segmented WBCs.

### Descriptions of data

The proposed method is tested on two different WBC image datasets. The first image dataset contains a total of 17,092 images of individual normal cells, which were acquired using the analyzer CellaVision DM96 in the Core Laboratory at the Hospital Clinic of Barcelona [30]. The dataset is organized into eight different groups. It includes neutrophils, eosinophils, basophils, lymphocytes, monocytes, immature granulocytes, erythroblasts, and platelets (thrombocytes). It is a high-quality labeled dataset that can be used for benchmarking, training, and testing models. They are JPG images with a size of (360$\times$363) pixels for each image. For more information refer to [30].

The second image dataset used in [31] contains 300 images together with the manually segmented images. The dataset was originally obtained from iangxi Tecom Science Corporation, China. The nuclei, cytoplasms, and background including red blood cells are marked in white, gray, and black respectively. The images were acquired using a Motic Moticam Pro 252A optical microscope camera with an N800-D motorized auto-focus microscope, and the blood smears were processed with a newly-developed hematology reagent for rapid WBC staining [31]. They are 120$\times$120 images of WBCs.

### Implementation and experimental results

#### Nucleus segmentation

**Fig. 6:**
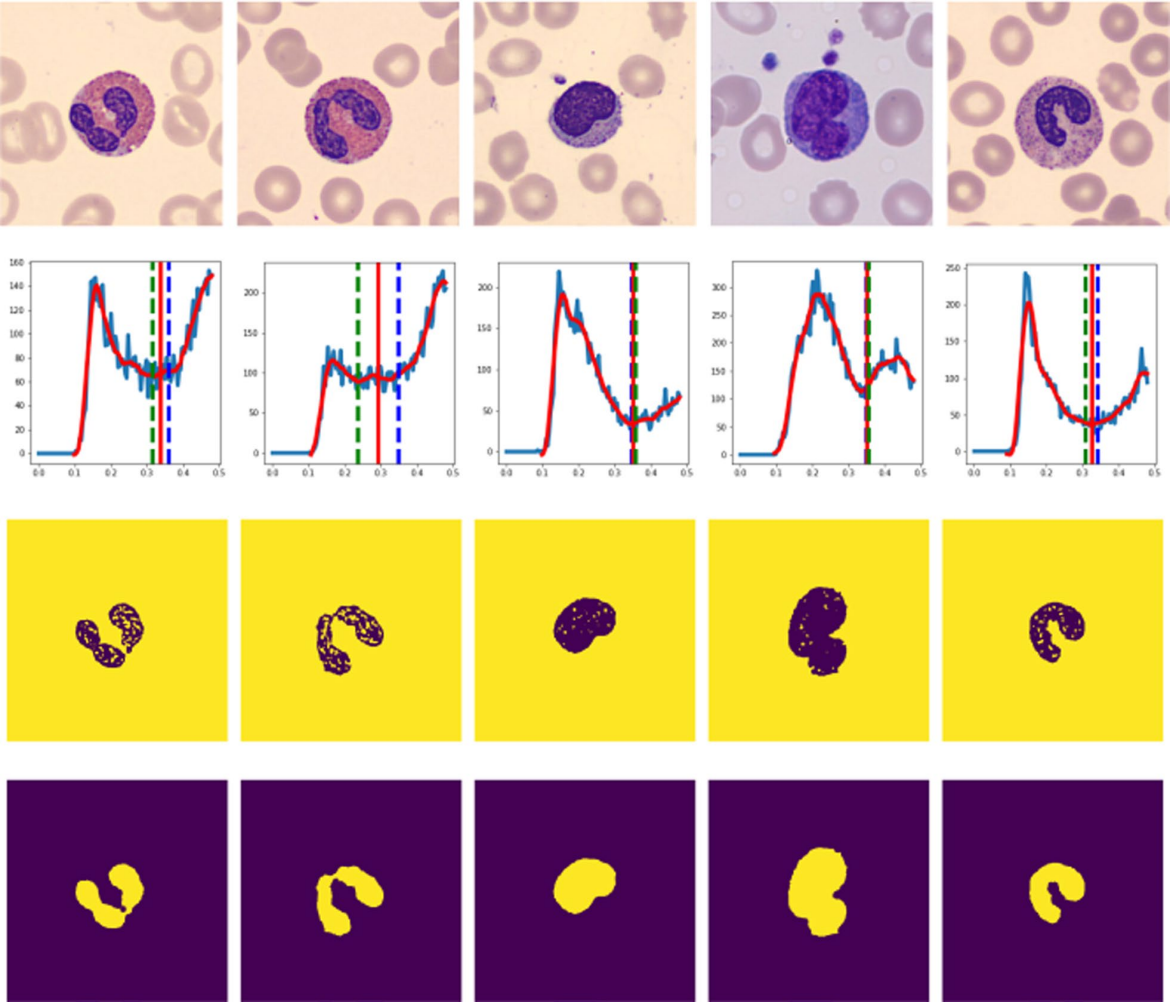
Nucleus segmentation steps. From the left, First row: Original images of eosinophils (first and second columns), lymphocyte (third column), monocyte (fourth column), and neutrophils (fifth column). Second row: Histogram decomposed and approximation. The dotted vertical lines indicated the values of $T_{nc0}$ and the local minimum respectively. The un-dotted vertical line indicates the estimated threshold ${\tilde{\varepsilon }}_{t}$. Third row: Binarized image using estimated threshold ${\tilde{\varepsilon }}_{t}$. Fourth row: Segmented nucleus after post-processing. The results were obtained by setting $Er= 0.07$

The input images are converted into grayscale. For each grayscale image, a value $T_{nc0}$ is computed using Eq. 4(a) in Additional file 1. Then, a *NumPy function histogram* is used for computing a histogram of the input image [32]. The histogram is decomposed into two equal parts and then run a script on the first part of decomposition to check and remove the long flat tail at the beginning (see Figs. 4 and 5). Then, a SciPy function *savgol filter* is applied to obtain the approximated function values [33]. A *B-spline* (3$^{rd}$ order polynomial) interpolation function is applied on the approximated function values and then the *argrelextrema* function is used to compute the local minima and maxima [33]. Note that if the local minima and maxima are not found in the first run, the length of decomposition is incremented, and start searching again until the first local maximum and minimum are found. The values of the first local maximum and minimum together with the value $T_{nc0}$ are used for computing the threshold ${\tilde{\varepsilon }}_{t}$ based on the conditions given in the methodology subsections. The threshold ${\tilde{\varepsilon }}_{t}$ is applied on the input image to preprocess and binarize it to segment the nucleus. The binarized image is post-processed by applying a morphological operation to remove small objects and close holes if they exist. The result is the segmented nucleus of the input image. Some results for the implementation steps are visualized in Fig. 6.

#### WBC and cytoplasm segmentation

**Fig. 7:**
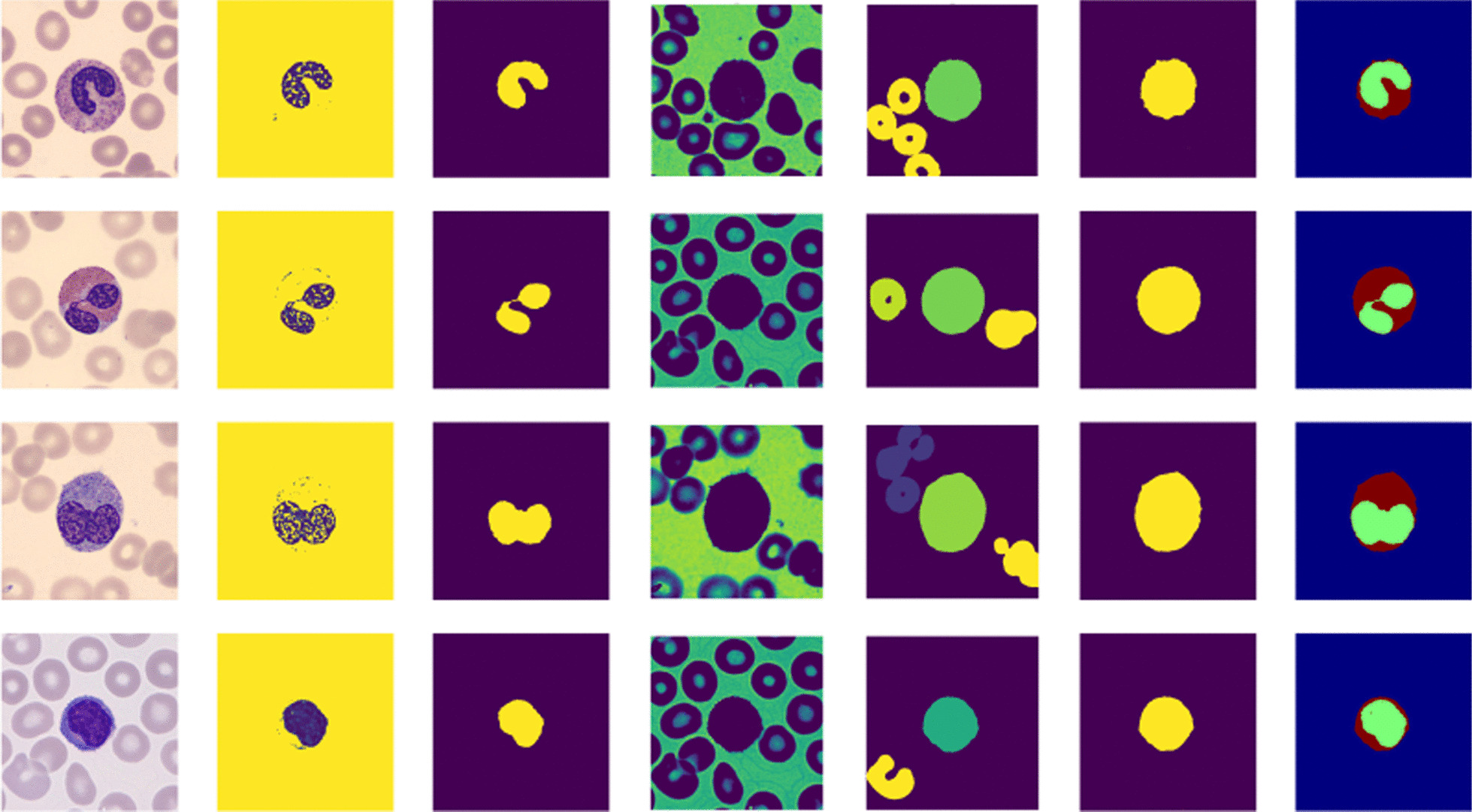
WBC and cytoplasm segmentation steps. From the left, First column: input images, Second column: Reconstructed images and then binarized using estimated threshold ${\tilde{\varepsilon }}_{t}$ for nucleus segmentation. Third column: Segmented nucleus after post-processing. Fourth column: Reconstructed images after averaging different thresholded images. Fifth column: Assigning new labels to the objects and removing some objects. Sixth column: Segmented WBC using a single-pixel value location from the segmented nucleus. Seventh column: Segmented nucleus and cytoplasm. From top to down: the first row is the neutrophil, the second row is the eosinophil, the third row is the monocyte, the fourth row is the lymphocytes

We generate *n* values automatically in the interval $[Min(A), \frac{Max(A)}{3}]$ which are referred as step size $er_{i}$ in Eq. 8. For each value in the interval we compute threshold $T_{wbc}^{i}$ using Eq. 8. Each threshold is applied to the input image to generate *n* thresholded images using Eq. 11. The *n* thresholded images are averaged using Eq. 12 to obtain a preprocessed image $A_{wbc}$ and then binarize it using a standard Otsu thresholding method [34]. Then, morphological operations are applied to separate the WBC from the background and RBCs. From the segmented nucleus (in the previous sub-section), we take a single pixel value location and use it to extract the whole WBC from the binarized image. The cytoplasm is obtained by subtracting the segmented nucleus from the WBC. Figure 7 visualizes some images summarizing the steps for nucleus and WBC segmentation.

For images whose WBCs touch the RBCs we opt for two classical approaches and use them to segment the WBCs.

**Fig. 8:**
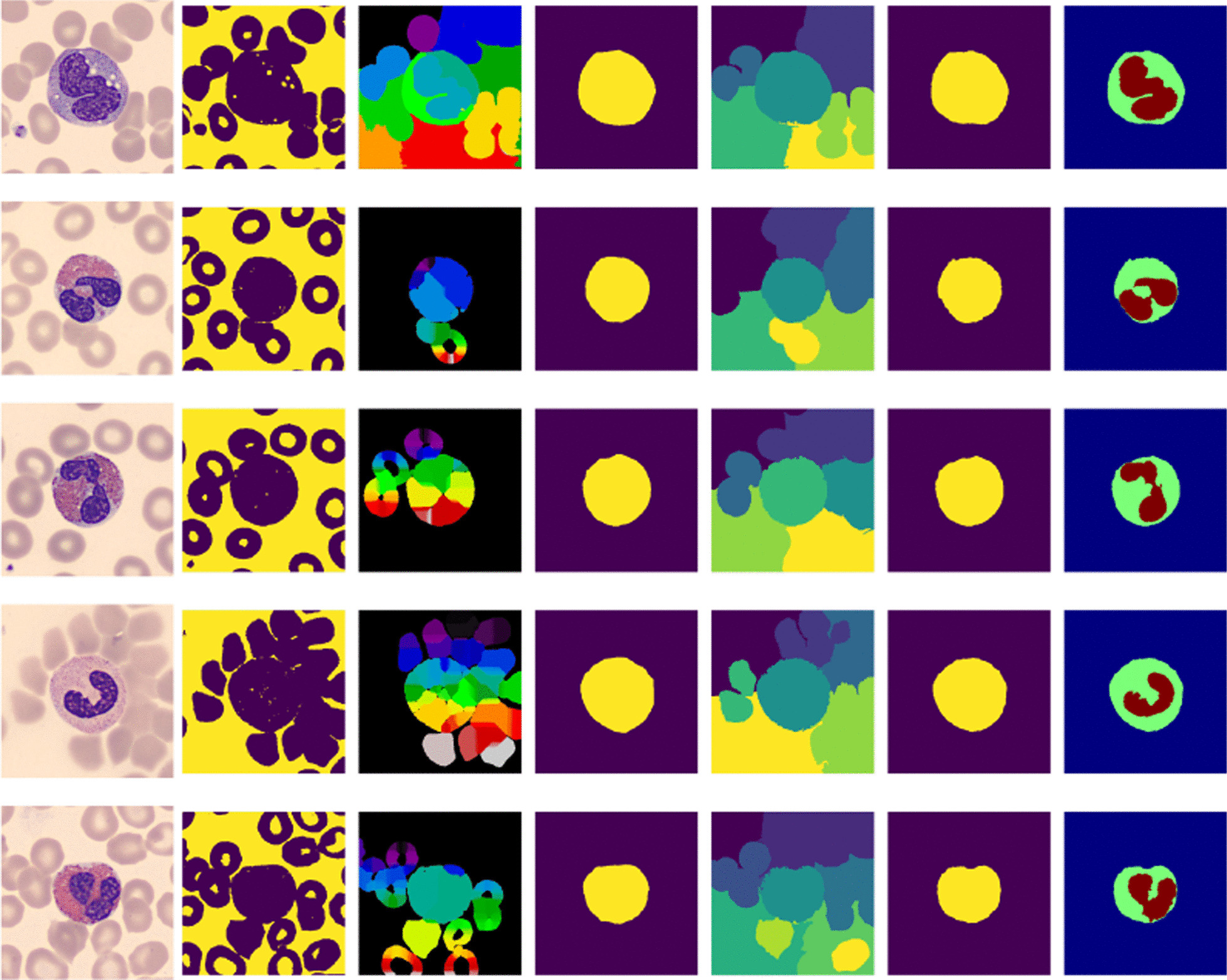
WBC segmentation using SLIC and marker-controlled watershed. From the left, First column: input images, Second column: converted into grayscale and then binarized using the Otsu thresholding method. Third column: marker-controlled watershed segmentation using a distance transformation. Fourth column: Segmented WBC after post-processing the watershed results. Fifth column: segmentation of the input image using SLIC. Sixth column: Segmented WBC from SLIC results. Seventh column: Segmented nucleus and cytoplasm

We apply the Simple Linear Iterative Clustering (SLIC) approach based on superpixel [26, 35]. Since the superpixel algorithm adheres to boundaries, we focus on identifying the WBCs boundaries whenever the RBCs touch the WBCs. We allow under segmentation in order to segment well the WBCs. The results are visualized in Fig. 8 fifth column.

We also apply watershed transformation for segmenting the WBCs when the RBCs touch the WBCs. The approach is a transformation on grayscale images [27]. It aims at detaching the ROI from the non interesting objects when the edges of these objects touch each other. We concentrate on the marker construction step where it is easier to control it. We allow an automatic choice of the number of local maxima and retain the results if the RBCs are detached from the WBCs. In this step, we do not care much about the over-segmentation of the ROI, instead, we focus on detaching the uninteresting objects from the object of interest (the WBC). The results are visualized in Fig. 8 third column.

Note that the input for watershed can be either image gradient or binarized image. Both options work but the challenging part is to control the markers. Looking at Fig. 8 third column it could be challenging to identify the required labels and then remove the unwanted ones. Figure 8 fifth column shows that SLIC is straightforward because it identifies the boundaries of the WBCs.

**Table 1:** Summary for 976 sample images (251 eosinophils, 201 neutrophils, 208 monocytes, and 316 lymphocytes)

	Eosinophils	Neutrophils	Monocyte	Lympoyctes
Thresholds mean for Nucleus segmentation	0.338991314	0.356037820	0.359911376	0.362329243
Thresholds mean for $\hbox {WBC}$ segmentation	0.894988532	0.887716863	0.892829014	0.890228957
Contrast Mean between nucleus and cytoplasm	0.555997217	0.531679043	0.532917638	0.527899714

### Quantitative analysis

We present a simple analysis to assess the efficiency and effectiveness of the proposed method. The performance evaluation is done by a quantitative measure of the segmentation results. We analyze the accuracy by evaluating the similarity between the segmentation results obtained using the proposed method and the ground truth.

We compute Jaccard Index (JI), Dice similarity coefficients (DSC), Sensitivity, Specificity, and Precision to check the level of similarity between the ground truth and the segmentation from the proposed method (predicted segmentation). The Jaccard Index (*JI*) is defined by

13 $$\begin{aligned} JI(L_{M}, L_{P})=\frac{|L_{M}(x,y)\cap L_{P}(x,y)|}{|L_{M}(x,y)\cup L_{P}(x,y)|} = \frac{TP}{TP+FP+FN} \end{aligned}$$

where $|L_{M}(x,y)|$ is the number of labels in the ROI from the ground truth. $|L_{P}(x,y)|$   is the number of labels in ROI obtained using the proposed method. $|L_{M}(x,y)\cap L_{P}(x,y)|$ is the number of labels appearing in the ROI from the ground truth and the predicted segmentation. The Dice Similarity Coefficient is defined by

14 $$\begin{aligned} DSC(L_{M}, L_{P})=\frac{2|L_{M}(x,y)\cap L_{P}(x,y)|}{|L_{M}(x,y)|+ |L_{P}(x,y)|} = \frac{2TP}{2TP+FP+FN} \end{aligned}$$

We can also use the concept of true positive (TP), false positive (FP), true negative (TN), and false-negative (FN) to check the performance of the method. We compute Sensitivity, Specificity, and Precision as follows.

15 $$\begin{aligned} Sensitivity = \frac{TP}{TP+FN}, \text { \textit{Specificity}} = \frac{TN}{TN+FP}, \text { Precision} = \frac{TP}{TP+FP} \end{aligned}$$

**Fig. 9:**
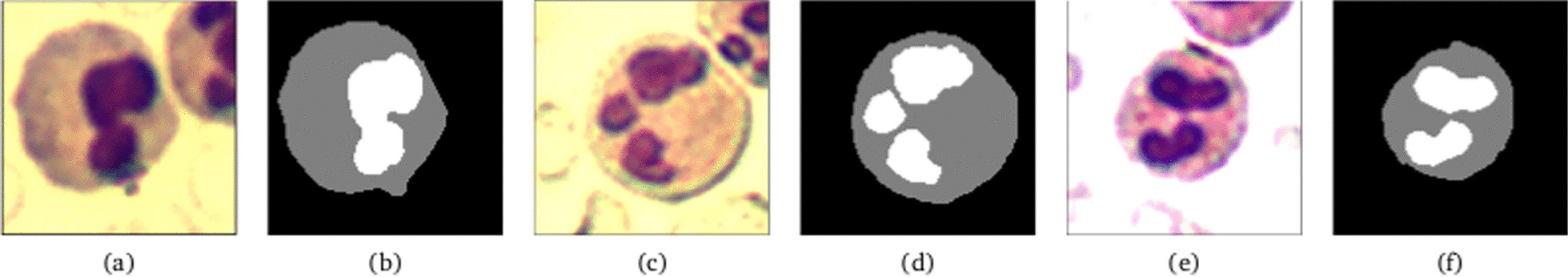
Sample of the excluded images. From the left: **a, c, e** are original images, whereas **b, d, f** are their corresponding ground truth images. Each original image seems to have two WBCs (one is showing only part of it) but the ground truth considered one cell which is at the center. Since the suggested approach is based on the intensity values it segments both WBCs

In the quantitative analysis we use the second data set used in [31] because the first data set in [30] does not have the ground truth images. In the analysis, some images were not included because of different reasons. Some of the images not considered in this section include the ones visualized in Fig. 9 showing the original and the ground truth,

A quantitative evaluation was performed by comparing the level of similarity between the ground truth and the segmentation obtained using the proposed method. The JI, DSC, Sensitivity, Specificity, and precision metrics range from 0 to 1. Zero indicates that there is no overlap between the predicted segmentation and the ground truth whereas 1 indicates a perfect overlap between predicted segmentation and the ground truth.

Also, we augmented 600 (masks included) images to generate 1860 images from the second data set. The data set for training, validation, and testing were divided in the ratio of 7:2:1 respectively. We use the U-net model for biomedical image segmentation presented in [36]. All the implementation were performed using Keras [37]. To see the setting of the hyperparameters, performance training of the model, and visualization of the predicted masks see Additional file 1. The prediction was performed using the testing data set (not included in the training). The predicted results are included in Tables 2 and 3. Other results obtained in other papers were also included for comparison.

**Table 2:** The performance of the proposed method for nucleus segmentation compared to U-Net, CNN, support-vector machine (SVM) based on the data set two presented using average in each measure of similarity

	JI	DSC	Specificity	Sensitivity	Precision
Proposed method	0.902925	0.948079	0.993110	0.952951	0.948903
U-Net [36]	0.917556	0.956456	0.995364	0.950505	0.964065
CNN [21]	–	0.910000	0.979200	0.960800	0.876300
SVM [21]	–	0.860000	0.818600	0.989700	0.934300

**Table 3:** The performance of the proposed method for WBC segmentation compared to deep neural network (LeukocyteMask(Aug-ET)), U-Net (implemented in [22] ) and WBC-Net based on the data set two presented using average in each measure of similarity

	JI	DSC	Specificity	Sensitivity	Precision
Proposed method	0.942908	0.970176	0.975164	0.992475	0.950133
U-Net [36]	0.941467	0.969628	0.989037	0.962368	0.977735
Deep Neural Network [22]	–	0.981960	–	–	0.995440
U-Net [22]	–	0.961630	–	–	0.933190
WBC-Net [23]	–	0.989200	–	–	0.989700

**Fig. 10:**
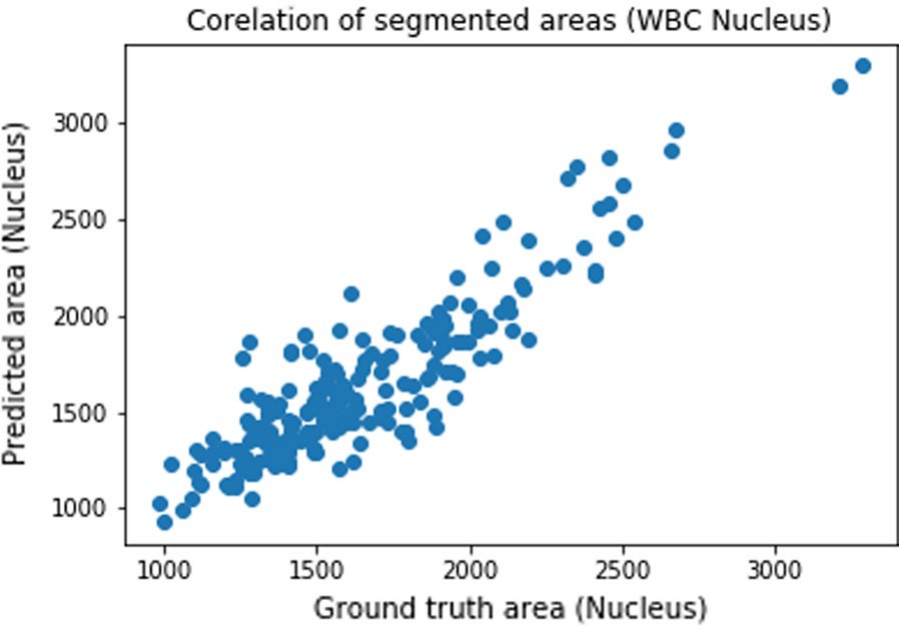
Correlation of the segmented nuclei size. Correlation for the size of the predicted nuclei to the size of the nuclei segmented manually

**Fig. 11:**
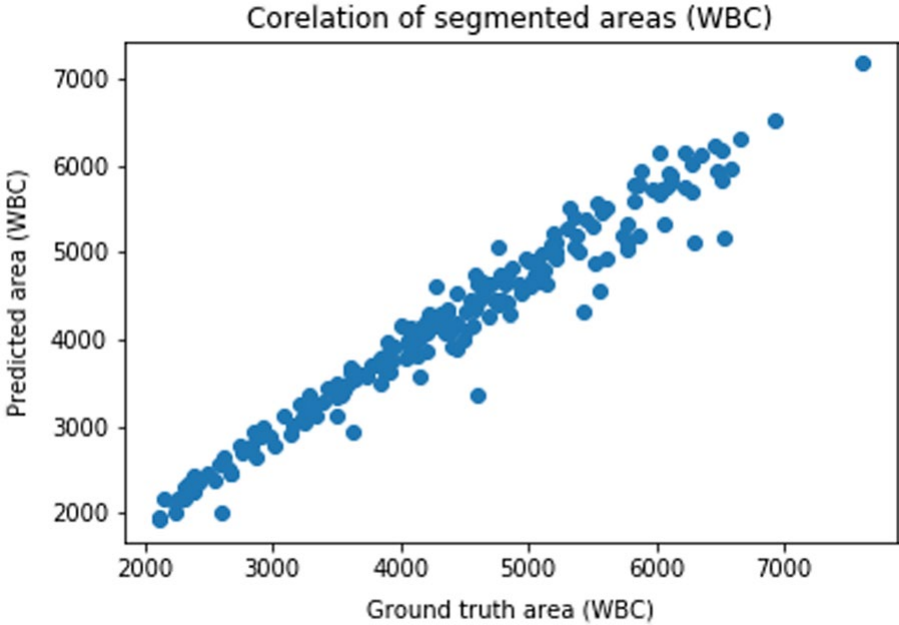
Correlation of the segmented WBCs size. Correlation for the size of the predicted WBCs to the size of the WBCs segmented manually

We also present graphs in Figs. 10 and 11 to show the correlation of the size of the segmented area (nucleus and WBC) obtained using the proposed method and the ground truth.

The implementation of the proposed method was done by writing scripts in the python programming language, and it was run on a PC processor (Core i7-8650U CPU @ 1.90GHz $\times$ 8 ). To process 40 images of 360 $\times$ 363 size, it takes 13.06518197059 seconds, which is approximately 0.326629549 seconds are used to process one image.

## Discussion and conclusion

We propose a method for segmenting the nucleus and cytoplasm of the WBCs based on the local minima. The method estimates the threshold automatically from the input image by checking different conditions that allow a wide range of searching for a good approximation. The threshold is applied to the input image to segment the nucleus. The WBC is segmented and then the cytoplasm is obtained by subtracting the segmented nucleus from the segmented WBC. The segmentation result is compared to ground truth to check the level of accuracy of the proposed method.

We segmented individual normal cells from the first data set which does not have ground truth. We also segmented images from the second dataset which has ground truth. The two data sets are significantly different from each other in terms of the colors of cytoplasm and background. But the results obtained from both data sets indicate that the proposed method can be applied to different image data sets. Figure 6 presents the results showing the nucleus segmentation steps whereas Fig. 7 presents the segmentation steps of cytoplasm and WBC. Figure 8 visualizes the results for cell segmentation using SLIC and marker-controlled watershed. Table 1 gives a summary of the threshold and contrast means for the WBCs.

We provide a summary of the performance analysis of the proposed method. We use Jaccard indices, Dice similarity coefficients, sensitivity, specificity, and Precision to evaluate the performance of the proposed method. The performance analysis results are summarized in Tables 2 and 3. We also compare the result obtained using the proposed method to the state of art methods. Table 2 compares the performance of the method on segmenting nucleus to the results obtained using CNN, and SVM [21]. Table 3 compares the performance of the method on segmenting WBCs to the results obtained using a Deep neural network [22], U-Net [22] and WBC-Net [23]. Also, we train the U-Net model in [36] on the augmented data and the results are included in Tables 2 and 3. Figure 10 provides a correlation of nuclei size between the predicted segmentation and the ground truth. Also, Fig. 11 gives a correlation of WBCs size between the predicted segmentation and the ground truth. Additional file 1: Figs. 12 and 13 in present the convergence of the normalized forward sensitivity index.

We performed a visual representation of segmentation results obtained using the proposed method. We provide more segmentation results in Additional file 1: Figs. 14 to 17. Also, Additional file 1: Fig. 18 provides subgraphs showing the impact of the control parameters by tuning different values. Additional file 1: Figure 19 shows the training performance, Additional file 1: Figs. 20 and 21 shows the prediction of the nuclei and WBC masks respectively. The proposed method has demonstrated its effectiveness in segmenting high-quality and poor-quality images. For images in which the RBCs do not touch the WBCs, the method works well even if the cytoplasm color is indistinguishable from the RBC’s color. For images in which the RBCs touch the WBCs, the method works well when the cytoplasm color is distinguishable from the RBCs. Whenever the cytoplasm color is indistinguishable from the RBCs and they touch each other, techniques for separating touching objects can be used. For images with very low contrast between the cytoplasm and background, the method can successfully segment the nucleus, but not the cytoplasm.

## Supplementary Information

**Additional file 1**. The file presents sensitivity analysis of parameters, Fig. 14 to 18 presentmore experimental results. Also, the file presents brie y theconcepts of B-Spline interpolation of the data points and the SLICalgorithm. Furthermore, the file presents results for theperformance of the U-Net model training and the predicted masks fornuclei and WBCs.

## Data Availability

All the data set analyzed in this study are freely available online. The first data set is available at “https://data.mendeley.com/datasets/snkd93bnjr/draft?a=d9582c71-9af0-4e59-9062-df30df05a121” and the second data set is available at is “https://github.com/zxaoyou”.

## Abbreviations

WBC: White blood cells
RBC: Red blood cells
SLIC: Simple linear iterative clustering
H and E: Hematoxylin and eosin
FCM: Fuzzy C- means algorithm
ALL: Acute lymphoblastic leukemia
RGB: Red, green, and blue
HSV: Hue saturation value
SVM: Support vector machine
ROI: Region of interest
JI: Jaccard index
DSC: Dice similarity coefficients
CIELAB: Implies L*a*b* color space, L* for perceptual lightness, a* and b* for the four unique colors of human vision
