# Supplementary material for: Threshold estimation based on local minima for nucleus and cytoplasm segmentation
Source: BMC Med Imaging. 2022 Apr 26;22:77. doi: 10.1186/s12880-022-00801-w (PMC9044622; doi:10.1186/s12880-022-00801-w)
Supplement: Supplementary file 1 — Additional file 1. The file presents sensitivity analysis of parameters, Fig. 14 to 18 presentmore experimental results. Also, the file presents brie y theconcepts of B-Spline interpolation of the data points and the SLICalgorithm. Furthermore, the file presents results for theperformance of the U-Net model training and the predicted masks fornuclei and WBCs. [file 12880_2022_801_MOESM1_ESM.pdf]

1. Supplementary Information

1.1. Sensitivity analysis of T_{nc0}

The concept of sensitivity analysis and parameter tuning for image segmentation has been used in [1]. We use the formula for normalized forward sensitivity index [2] of the variable T_{nc0} to a parameter n which is defined as

$$\Upsilon_{n}^{T_{nc0}} = \frac{\partial T_{nc0}}{\partial n} \times \frac{n}{T_{nc0}} \tag{1a}$$

From Eq. 2 in the article. For n_1

$$\Upsilon_{n_1}^{T_{nc0}} = \frac{\partial T_{nc0}}{\partial n_1} \times \frac{n_1}{T_{nc0}} = -\frac{Max(A)}{n_1^2} \times \frac{n_1}{T_{nc0}} \tag{2a}$$

For n_2

$$\Upsilon_{n_2}^{T_{nc0}} = \frac{\partial T_{nc0}}{\partial n_2} \times \frac{n_2}{T_{nc0}} = -\frac{Min(A)}{n_2^2} \times \frac{n_2}{T_{nc0}} \tag{3a}$$

By considering a series of images we investigate the convergence of $\Upsilon_{n_1}^{T_{nc0}}$ and $\Upsilon_{n_2}^{T_{nc0}}$ as $n_1 \to \infty$ and $n_2 \to \infty$ respectively.

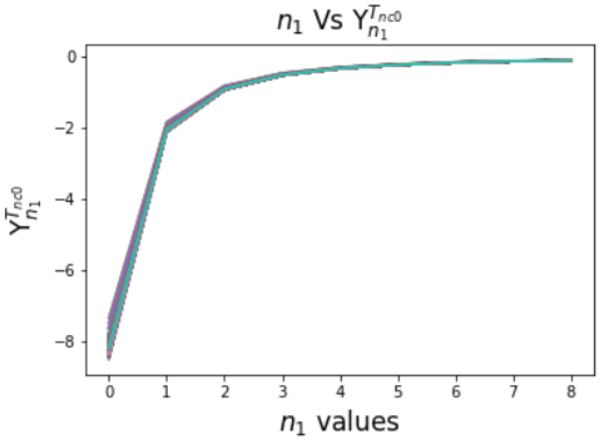

Figure 12: The values of $\Upsilon_{n_1}^{T_{nc0}}$ start stabilizing around $n_1 = 3$. The number of images tested is 8960.

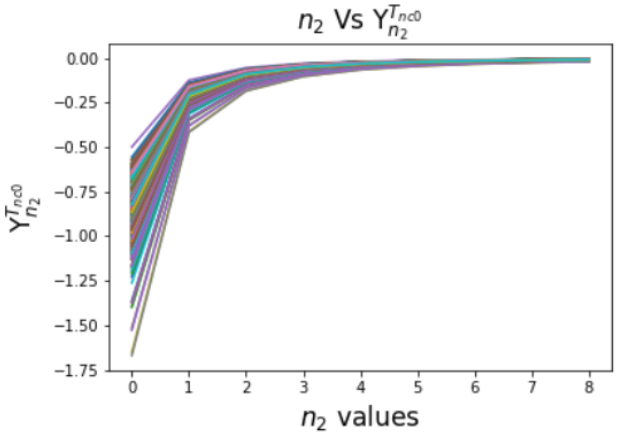

Figure 13: The values of $\Upsilon_{n_2}^{T_{nc0}}$ stabilizes around $n_2 = 3$. The number of images tested is 8960.

For $n_1 = n_2 = 3$, Eq. 2 in the article becomes

$$T_{nc0} \approx \frac{Max(A)}{3} + \frac{Min(A)}{3} \tag{4a}$$

From Eq. 4a, for images whose intensity values are in the intervals $[Min(A), Max(A)] = [0, 1]$, $T_{nc0} = \frac{1}{3}$.
For images whose intensity values are in the interval of either $(Min(A), Max(A)) = (0, 1)$, $(Min(A), Max(A)] = (0, 1]$

or $[Min(A), Max(A)) = [0, 1)$, either $T_{nc0} \leq \frac{1}{3}$ or $T_{nc0} \geq \frac{1}{3}$.
Looking at Fig. 12 and 13, the values of $\Upsilon_{n_2}^{T_{nc0}}$ and $\Upsilon_{n_1}^{T_{nc0}}$ show that n_1 is more sensitive compared to n_2. So, n_1 needs to be chosen carefully.

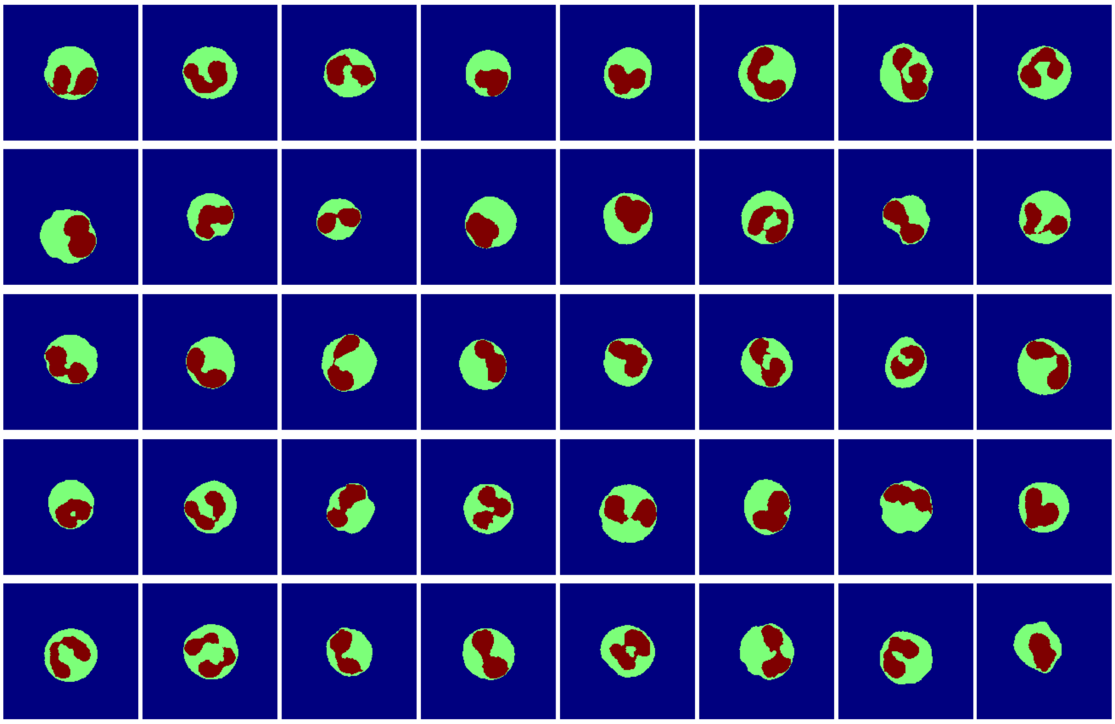

Figure 14: Segmented nucleus and cytoplasm of white blood cells. Eosinophil images.

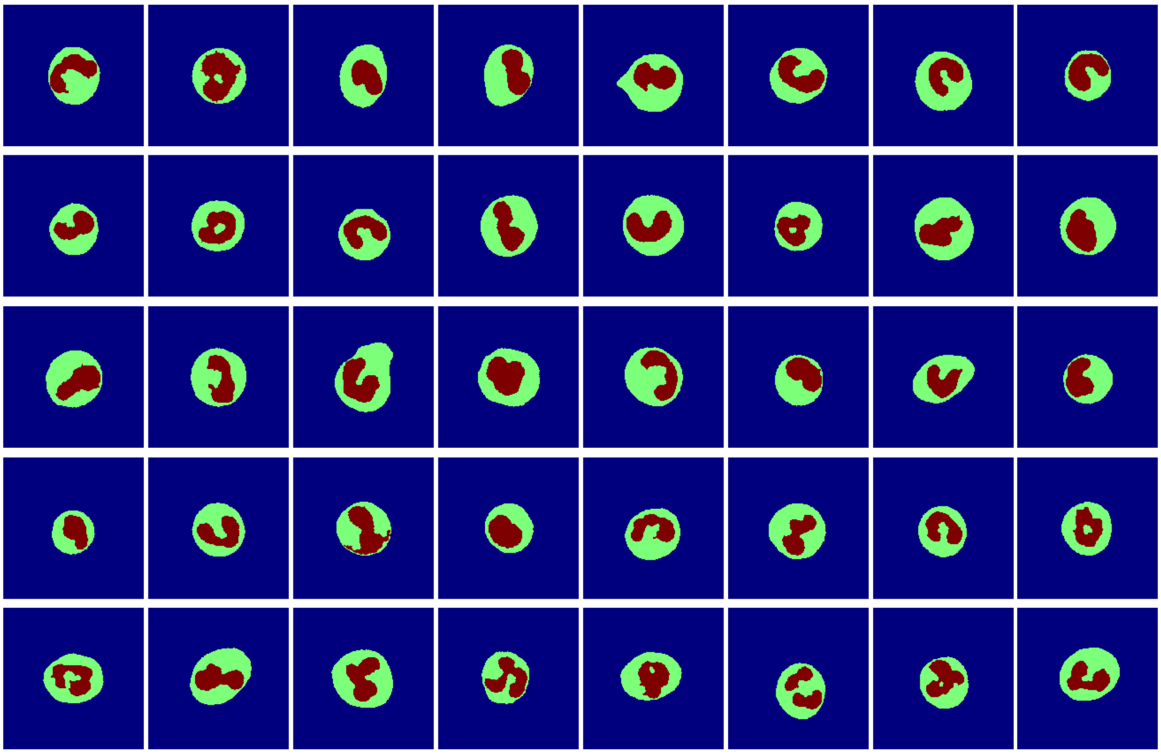

Figure 15: Segmented nucleus and cytoplasm of the white blood cells. Neutrophils images.

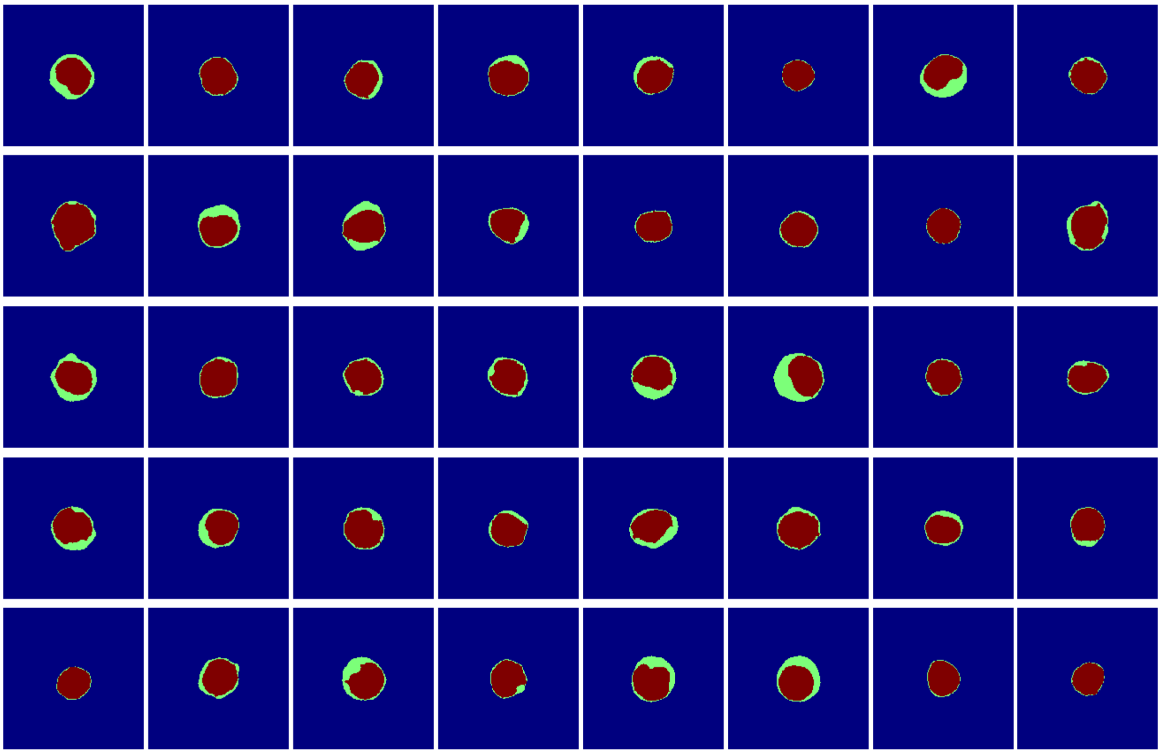

Figure 16: Segmented nucleus and cytoplasm of white blood cells. Lymphocytes images.

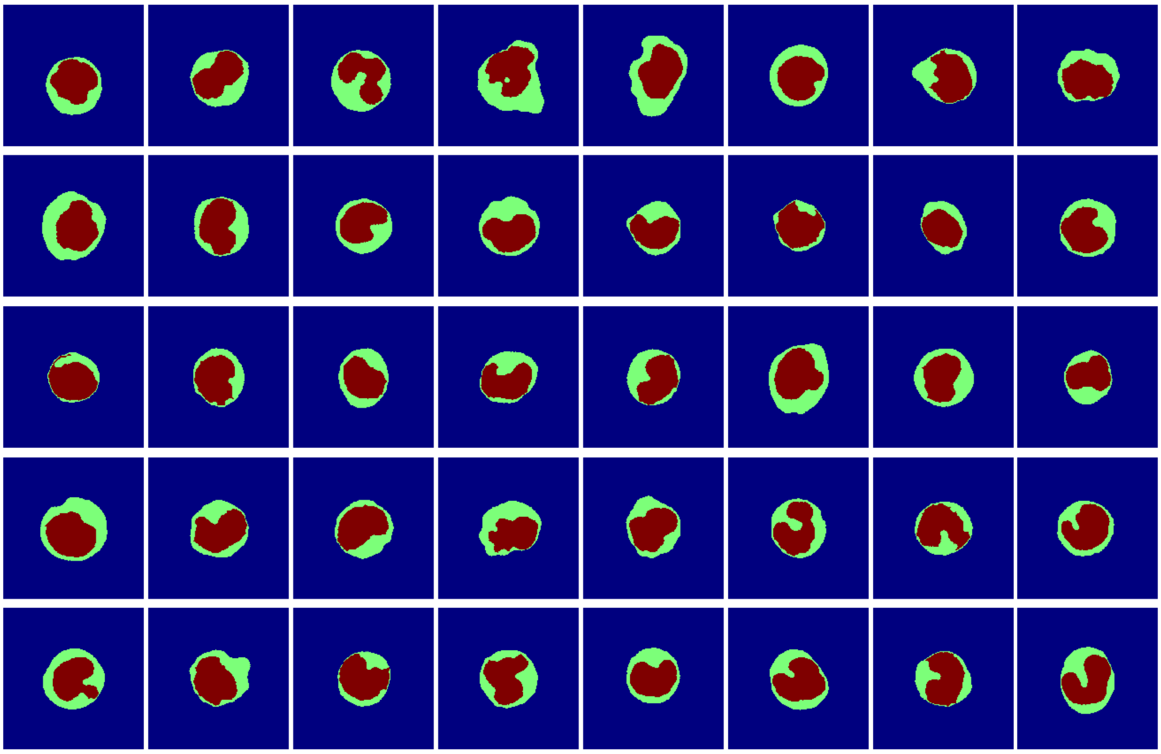

Figure 17: Segmented nucleus and cytoplasm of the white blood cells. Monocyte images.

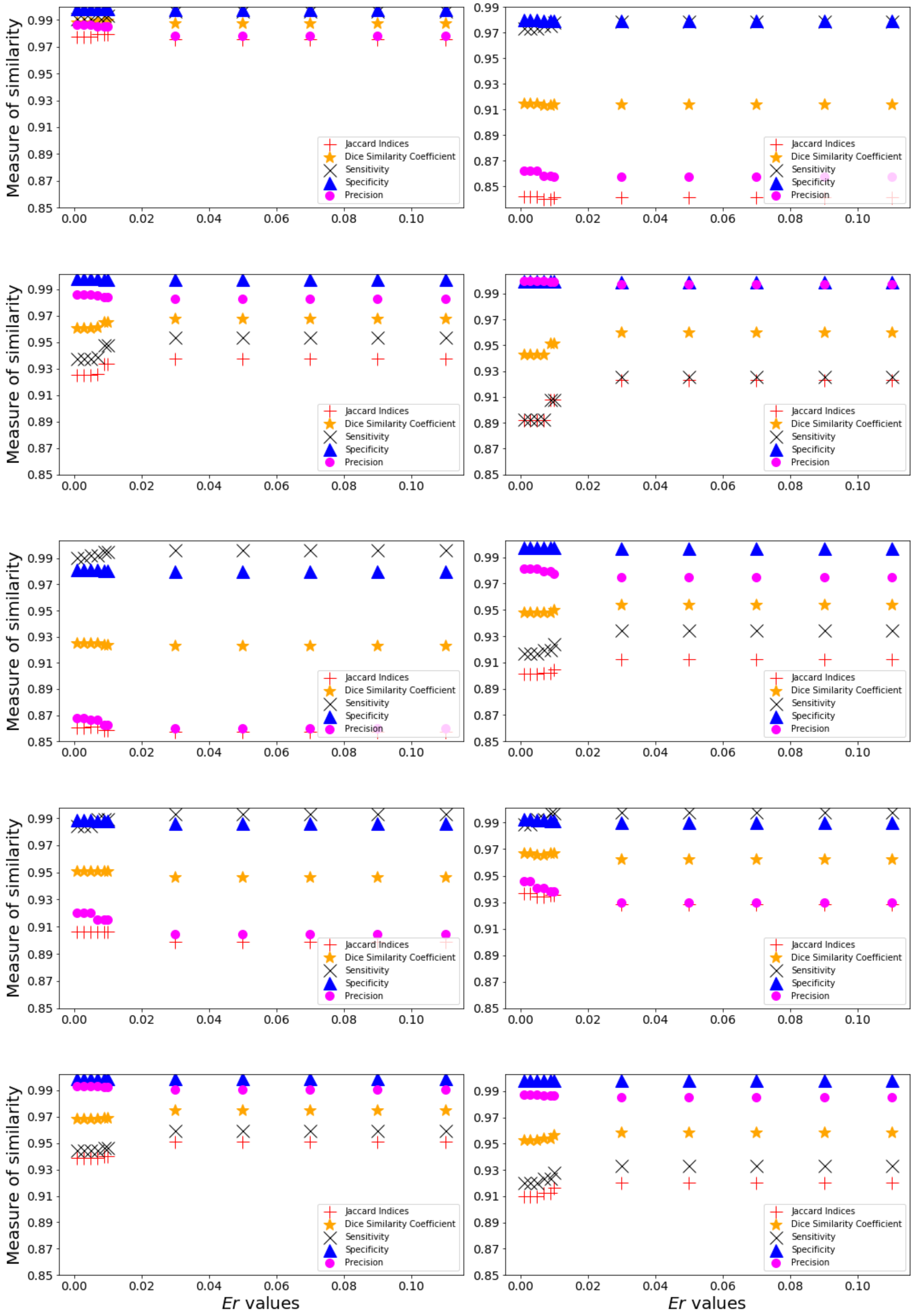

Figure 18: Graphs showing measures of similarity (vertical axis) of the segmented nucleus using the proposed method in relation to the tuned Er values (horizontal axis). The Er values tested include: 0.001, 0.003, 0.005, 0.007, 0.009, 0.01, 0.03, 0.05, 0.07, 0.09, 0.11.

1.1.1. B-Spline interpolation of the data points

A spline is a piecewise polynomial function that is constructed as a means to interpolate a set of data points . Each piece of the function is confined between knots $t_i < t_{i+1}$ with strict continuity conditions between each piece at the knots. These polynomials are strung together at the knots to form the completed curve . Now, B-splines (basis splines) are a set of piecewise functions that form a basis for the set of all possible spline functions. Any spline function can be described as a linear combination of B-splines.

Let U be a set $m+1$ non-decreasing numbers $t_0 < t_1 < t_2 < \cdots < t_m$ over the interval which we want to interpolate the data. The t_is are called knots and the set U is called knot vector. Then, the ith B-spline function of degree 0 is given by:

$$B_i^0(x) = \begin{cases} 1 \text{ if } t_i < x < t_{i+1} \\ 0 \text{ elsewhere} \end{cases}$$

The subsequent higher-level B-splines are constructed from the recurrence relation:

$$B_i^d(x) = \frac{x - t_i}{t_{i+d} - t_i} B_i^{d-1}(x) + \frac{t_{i+d+1} - x}{t_{i+d+1} - t_{i+1}} B_i^{d-1}(x) \tag{5a}$$

Equation 5a creates the basis functions for a d degree spline. So, a linear combination of B-Splines or spline function is a combination of form

$$g(x) = \sum_i^n c_i^d B_i^d(x) \tag{6a}$$

where c_is are the coefficients of the basis functions. The computation of these coefficients can be found in the reference sources. A linear combination of these functions in equation 6a can be used to interpolate a data set. In order to interpolate the data, the B-spline curve must span the interval of the data points to be interpolated.

1.1.2. Summary of SLIC Algorithm

Mainly, the algorithm involves three steps which include initialization, assignment, and enforce-connectivity. It performs K-means segmentation using 5 dimension space of color information and image location. The RGB color space is transformed into $CIELAB$ color space [3] and then each of the pixel images is mapped into (l, a, b, x, y). The algorithm starts by initializing a k number of clusters which are sampled on a regular grid. Then in the assignment step each pixel p_i is associated with the nearest cluster centre. Different from the standard k-means, SLIC updates the cluster centres by searching in a certain neighbourhood and not on the whole image. But the update step depends on the convergence of the error term which is obtained by computing the residual error between the old and the new centres. In case there are disjoint pixels from the previous steps then enforcing connectivity is applied so as to assign them a nearest superpixel.

1.2. Training U-Net Model for segmenting Nucleus and WBCs

The model training was run on google colab [4] using NVIDIA-SMI TESLA k80. The model was trained by using activation 'relu', kernel initializer 'he_normal', and padding is the same. The activation function used is sigmoid and the initial learning rate is set to be 0.0001, and we use the Adam optimizer. The training performance is visualized in Figure 19. The predicted nuclei and WBCs masks are visualized in Figure 20 and 21 respectively.

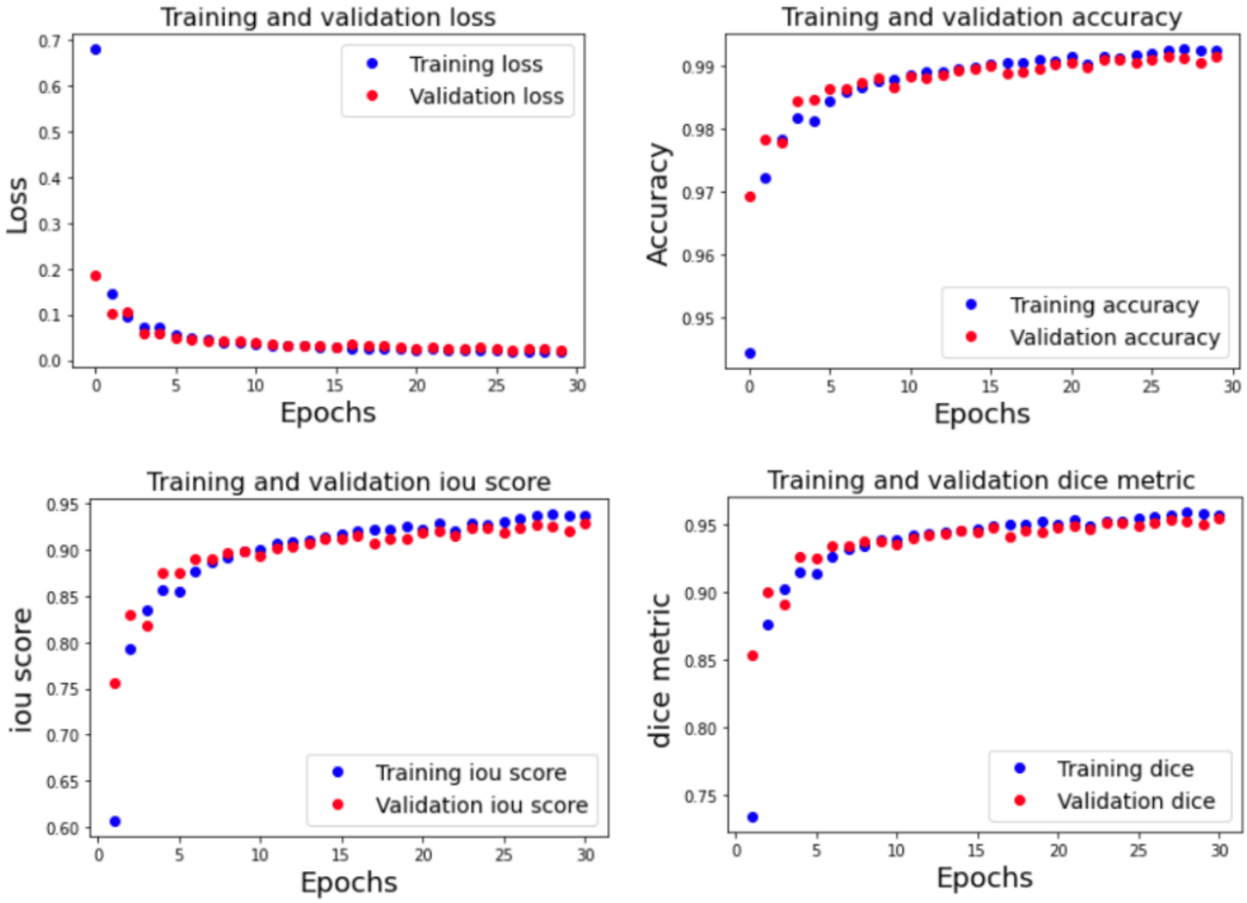

Figure 19: Training performance for predicting nuclei masks. First row: Loss and accuracy for both training and validation. Second row: Dice similarity coefficient and iou score during the training and validation. The results for performance training of the WBCs masks is not included.

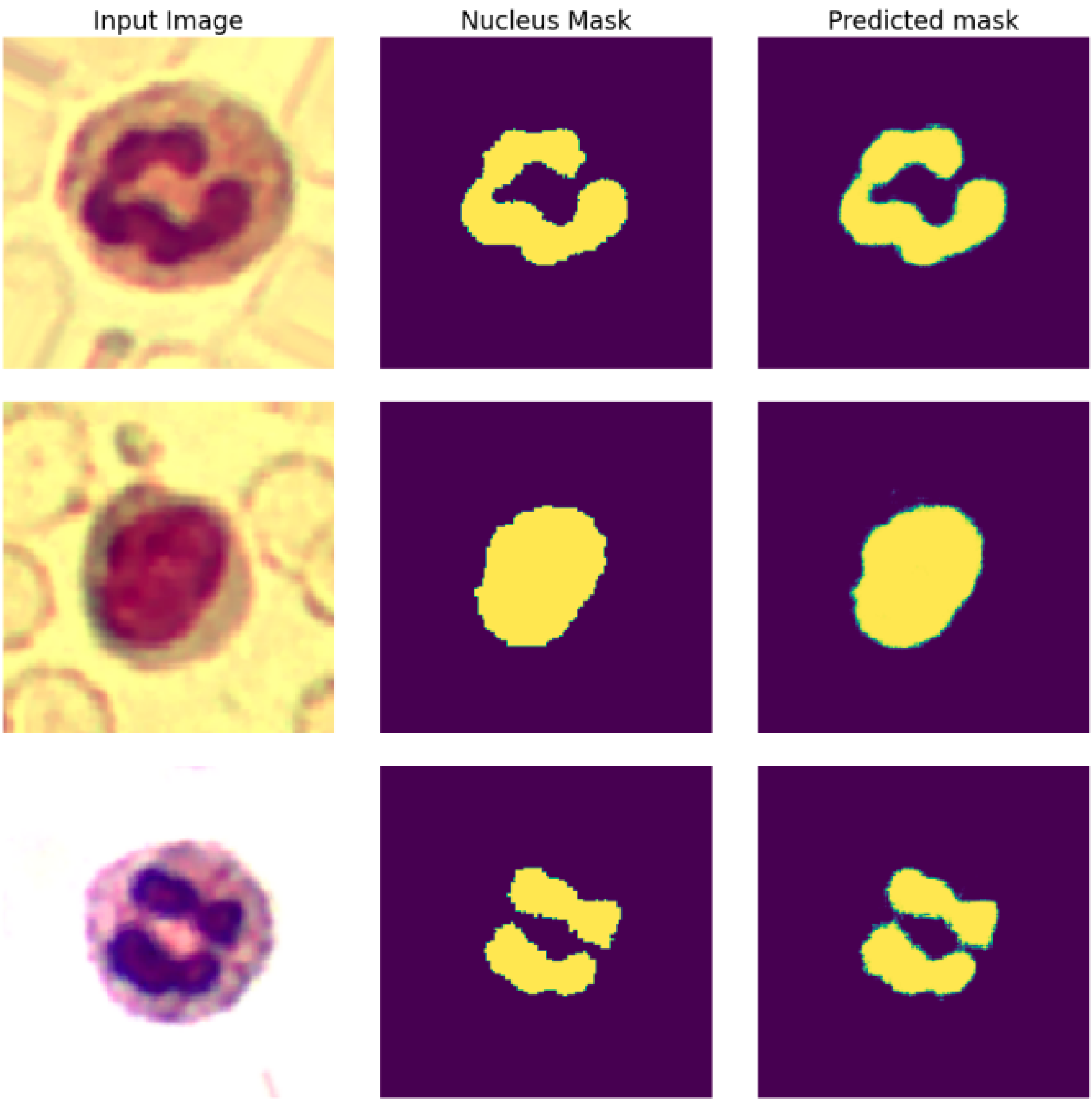

Figure 20: Prediction of nuclei masks: The first column presents input images, the second column presents the nuclei masks, and the third column shows the predicted nuclei masks.

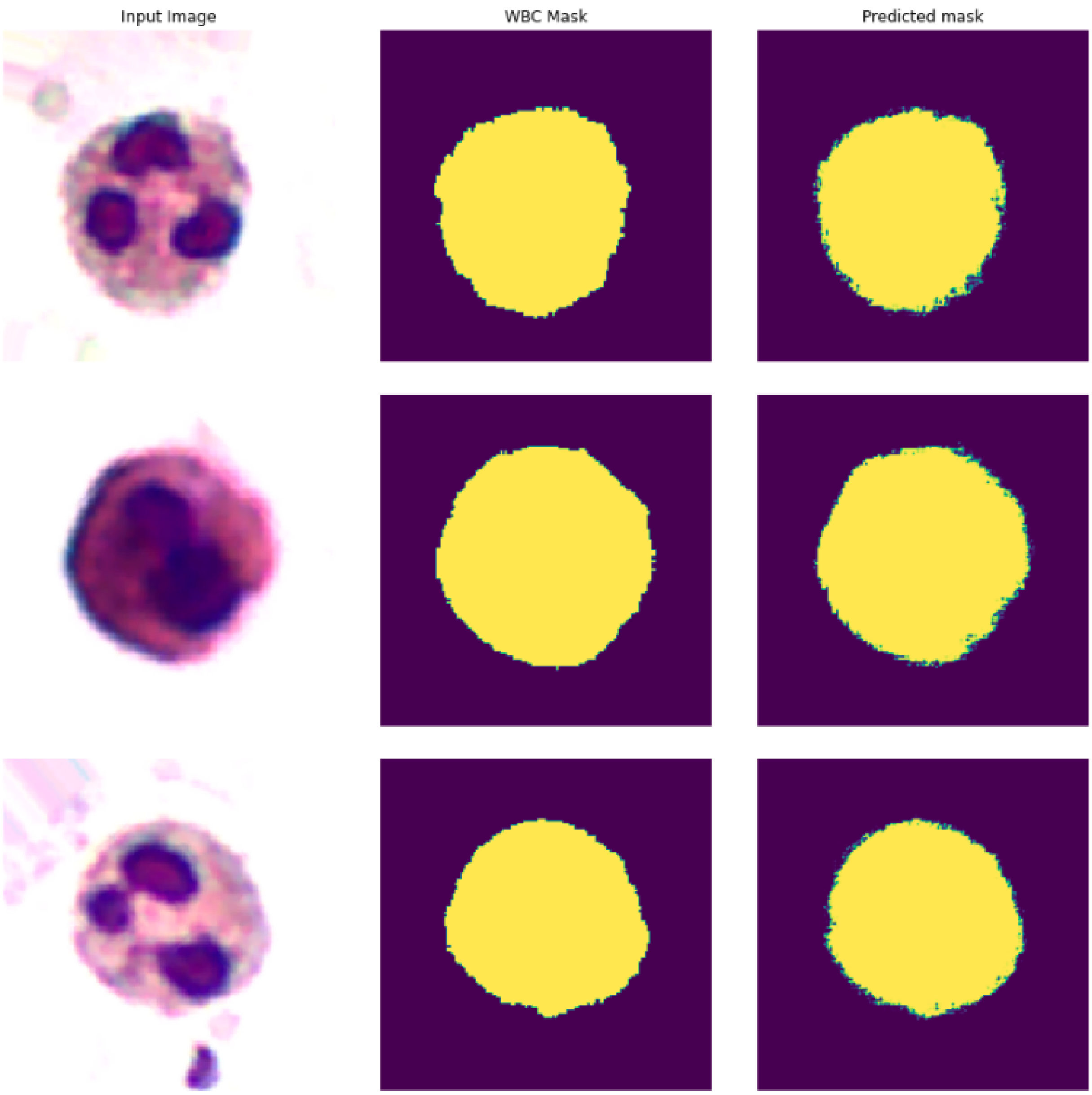

Figure 21: Prediction of WBCs masks:The first column presents input images, the second column presents the WBCs masks, and the third column shows the predicted WBCs masks.

References

[1] G. Teodoro, T. M. Kurç, L. F. Taveira, A. C. Melo, Y. Gao, J. Kong, and J. H. Saltz, "Algorithm sensitivity analysis and parameter tuning for tissue image segmentation pipelines," *Bioinformatics*, vol. 33, no. 7, pp. 1064–1072, 2017.

[2] N. Chitnis, J. M. Hyman, and J. M. Cushing, "Determining important parameters in the spread of malaria through the sensitivity analysis of a mathematical model," *Bulletin of mathematical biology*, vol. 70, no. 5, p. 1272, 2008.

[3] C. Pub, "Technical report 15: 2004: Colorimetry," 2004.

[4] E. Bisong, *Building machine learning and deep learning models on Google cloud platform*. Springer, 2019.
